# MGGAN: A multi-generator generative adversarial network for breast cancer immunohistochemical image generation

**DOI:** 10.1016/j.heliyon.2023.e20614

**Published:** 2023-10-05

**Authors:** Liangliang Liu, Zhihong Liu, Jing Chang, Hongbo Qiao, Tong Sun, Junping Shang

**Affiliations:** College of Information and Management Science, Henan Agricultural University, Zhengzhou, Henan 450046, PR China

**Keywords:** Immunohistochemical techniques, Hematoxylin and eosin, Generative adversarial network, Multi-generator, Patch-based discriminator

## Abstract

The immunohistochemical technique (IHC) is widely used for evaluating diagnostic markers, but it can be expensive to obtain IHC-stained section. Translating the cheap and easily available hematoxylin and eosin (HE) images into IHC images provides a solution to this challenge. In this paper, we propose a multi-generator generative adversarial network (MGGAN) that can generate high-quality IHC images based on the HE of breast cancer. Our MGGAN approach combines the low-frequency and high-frequency components of the HE image to improve the translation of breast cancer image details. We use the multi-generator to extract semantic information and a U-shaped architecture and patch-based discriminator to collect and optimize the low-frequency and high-frequency components of an image. We also include a cross-entropy loss as a regularization term in the loss function to ensure consistency between the synthesized image and the real image. Our experimental and visualization results demonstrate that our method outperforms other state-of-the-art image synthesis methods in terms of both quantitative and qualitative analysis. Our approach provides a cost-effective and efficient solution for obtaining high-quality IHC images.

## 1 Introduction

Breast cancer (BC) is the second-most prevalent cancer and the leading cause of death for females worldwide [1]. In the United States and the European Union, approximately 10% of women are at risk of developing BC [2], while in China, the figure is 24.2% [3]. BC patients have a mortality rate of 10% to 20% in the early stages, which increases to 90% in the late stages [4], [5]. Therefore, screening for BC is a necessary means to prevent its development.

Histopathology is the gold standard for diagnosing BC, but it is a tedious process. First, doctors need to make living breast tissue materials into hematoxylin and eosin (HE) staining sections. Then, experienced pathologists diagnose breast tissue by observing the HE section under a microscope or analyzing digitized whole-section images (WSI). In order to further accurately diagnose BC, diagnostic markers derived from the molecular phenotype and histological subtype of biopsy tissue slices, such as human epidermal growth factor receptor 2 (HER2), must be determined [6], [7]. Finally, the pathologist determines the tumor grade and formulates the corresponding treatment plan through the correct analysis of HER2 expression. Obtaining the correct HER2 expression is key to diagnosis. Currently, the immunohistochemistry technique (IHC) is the primary method used to evaluate HER2 expression. IHC is a routine technology for evaluating diagnostic markers, analyzing biopsy tissue sections, and determining the molecular phenotype and histological subtype of tissue sections [8]. It is widely used to identify metastatic and benign tumors and grade breast cancer [9]. To our knowledge, traditional IHC staging technologies are a complex and labor-intensive task. For example, the IHC staging systems proposed by McCarty et al. [10] and Allred et al. [11] are limited by manual time consumption and accuracy, while commercial IHC staging systems are limited by high equipment and software costs [12]. How to obtain sections at a lower cost while ensuring the quality of IHC-stained sections is a challenge for cancer diagnosis. A potential solution to this challenge is translating the cheap and easily available HE slices into IHC slices.

### 1.1 Related work

Translating a HE slice into an IHC slice is an image-to-image translation (I2I) task. I2I is used to translate an image from the source domain to the target domain while retaining its content representation. It is widely used in natural image synthesis tasks [13], [14]. With the development of depth learning technology, I2I depth-learning-based I2Is were widely used in various fields such as image generation, image segmentation, image restoration, pose prediction, and other fields [15], [16], [17]. In this section, we will review the existing I2I work. In the past few years, several methods have been developed to solve the problem of image-to-image translation by using generation models. At the same time, we also describe the challenges that remain unresolved in I2I research.

In general, there are two types of I2I algorithms: unsupervised and supervised. The unsupervised image translation method does not require paired datasets. Unsupervised strategy-based image translation research is widely used as it can learn the mapping of two domains without paired images during training. The unsupervised-based image translation methods find the mapping between source domain images and target domain images from unpaired training data, finally finish image translation and generate the synthetic image. Such methods are widely used in image translation tasks. An unsupervised generative adversarial network-based (GAN) method (DiscoGAN) was proposed for recognizing relations between data from different domains on unpaired data [18]. DiscoGAN successfully transferred images from one domain to another while preserving the key attributes of images. Huang et al. [19] developed an unsupervised image-to-image conversion framework called MUNIT. This framework represents images with domain-invariant content codes and captures domain specific attribute style codes. It converts images to another domain by recombining the content code with the style code sampled from the style space of the target domain, finally realizing the generation of multi-mode output from a given source domain image. In addition, Liu et al. [20] proposed an image-to-image translation framework based on the coupled GAN in an unsupervised manner. The proposed GAN presented high-quality image translation results on various challenging unsupervised image translation tasks. Inspired by dual learning, Li et al. [21] developed a dual-GAN network (DualGAN) to image synthetic tasks. DualGAN used multi-source datasets and an unsupervised training strategy to synthesize prediction image from the original image. Moreover, the proposed image synthetic error loss function improved the performance of the DualGAN method. Zhu et al. [22] introduced the cyclic consistency loss into GAN and proposed a new GAN (Cycle GAN), which has obtained an advanced level in multiple unpaired datasets. However, unsupervised methods are inherently more challenging than supervised methods since they lack corresponding outputs. Additionally, the absence of labeled input data may result in inaccurate results, which requires further improvements in the accuracy of such methods.

To overcome these challenges, some paired GAN works have been introduced in I2I tasks. One of the most prominent approaches is Pix2pix [15]. Pix2pix is an improved GAN method, which is the first application of the conditional GAN to solve the I2I task in a supervised manner. Subsequently, Wang et al. [23] put forward a differentiated area proposal countermeasure network (DRPANs). DRPANs solved the problem of fuzzy prediction results caused by pixel loss in the original Pix2pix model by incorporating modifiers. In addition, based on Pix2pix, AlBahar et al. [24] proposed an improved GAN (pix2pixHD). The Pix2pixHD model uses a multi-generator and a discriminator to generate high-resolution images. Furthermore, the pix2pixHD model can produce different outputs for the same input. Moreover, SRGAN [25] incorporates a spatially-adaptive normalization layer into the GAN structure, this change helps the original GAN synthesize photo-level photo-realistic images under the semantic layout as the input. CoCosNet [16] established a dense semantic correspondence between cross-domain images by constructing an intermediate domain as a bridge, and then synthesized images by incorporating the appearance of semantic corresponding patches. And beyond that, PixelNN [26], ASAPNet [27], and CoCosNetv2 [28] applied the GANs to image super-resolution tasks. These models usually were used in natural image translation tasks. They take semantic information as input and translate it into real images. These models train the semantic information of objects or natural images as input under supervised strategies and then realize image conversion.

Medical images differ significantly from natural images due to the challenges of missing or blurring multi-modal images. However, image translation research offers a promising solution to this problem. For instance, Cho et al. [29] proposed a conditional GAN-based Stain Style Transfer (SST) model for the translation of histopathological stain images to aid in tumor classification. Shaban et al. [30] introduced a CycleGAN-based model to correct the abnormal staining of digital tissue images. Additionally, the Pyramid Pix2Pix method, presented by Liu et al. [31], was developed to translate HE images to IHC images for the standardization and translation of pathological image dyeing. Nonetheless, I2I-based image translation research is rapidly advancing in the medical image field, and further improvements in its performance are needed.

### 1.2 Contributions

In order to promote research in the medical imaging field, we propose a multi-generator GAN (MGGAN) for translating HE image into immunohistochemical image with a fucus on BC. The main contributions of our proposed MGGAN are outlined below:

1. We propose a multi-generator GAN (MGGAN) to generate an IHC image based on the HE image of BC. MGGAN adopts the low-frequency and high-frequency components of the image to improve the translation of BC image details.

2. In MGGAN, the U-shaped architecture-based generators solve low-frequency components, the patch-based discriminator solves high-frequency components.

3. We incorporate the cross-entropy loss as the correct regularization term into the loss function to correct the consistency between the synthetic image and the real image.

4. The compaction results and extensive visual experiments demonstrate the proposed MGGAN achieves better translation results on a public challenge BCI dataset.

The remainder of this paper is organized as follows. Section 2 describes the methodology of the proposed MGGAN and the quantitative evaluations. Section 3 introduces the material and data preparation. Section 4 presents the results of empirical comparative and ablation experiments that investigate the contributions of the individual components of the proposed network. Finally, we discuss and summarize the results of this study in Sections 5 and 6, respectively.

## 2 Methodology

### 2.1 GAN for I2I

GAN represents one of the most common neural network in the I2I field [15], [32]. A typical GAN consists of two sub-networks: a generator (G) and a discriminator (D). G receives input image (*x*) and generate transformed image ($\overset{ˆ}{y}=G(x)$). G usually adopts supervised training methods. D distinguishes whether the generated image $\overset{ˆ}{y}$ is a real image or a fake image. D is trained to distinguish between the translated $\overset{ˆ}{y}$ and target *y*, and G is trained to generate $\overset{ˆ}{y}$, so that the transformed $\overset{ˆ}{y}$ is infinitely close to the target *y*. The ultimate goal is that D cannot distinguish $\overset{ˆ}{y}$ from *y*. The process of GAN learning is the process of G and D subnets adversarial training, which can be expressed by the following formula:

(1) $$min_{G}min_{D}E_{∽p_{target}}[\log D(y)]+E_{x∽p_{x}}[1-\log D(G(x))],$$

where *x* and $p_{x}$ denote the input image and its distribution, *y* is the expected value. Some GANs also take the random noise as a part of inputs and introduce the random noise to make the translated images diversified. Due to the distance between the decision boundary of D and $\overset{ˆ}{y}$ is hardly considered by the noise, it may lead to the instability of the learning process of D. To overcome this problem, Mao et al. [33] introduced the least squares function into D in 2017, which stabilized the learning process of D.

Although the introduction of random noise makes the translated image diverse, Pix2pix GAN indicates that the distribution of the generated images in image translation depends on the distribution of the source images. Therefore, Isola et al. [15] introduced conditional GAN into the image translation task. Specifically, they introduced the least squares function and conditional input in Eq. (1). The loss functions L(D) (Eq. (2)) and L(G) (Eq. (3))of D and G are expressed as follows:

(2) $$L(D)=\frac{1}{2}E_{y∽p_{target}}[{\left(D(x,y)-1\right)}^{2}]+\frac{1}{2}E_{x∽p_{x}}[D{\left(x,G(x)\right)}^{2}],$$

(3) $$L(G)=\frac{1}{2}E_{x∽p_{x}}[{\left(D(x,G(x))-1\right)}^{2}].$$

### 2.2 Overview of our proposed architecture

**Figure 1:**
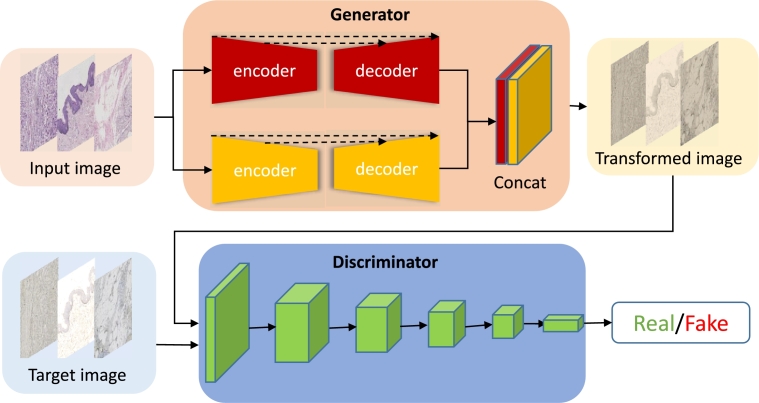
Overview of the MGGAN architecture. The high-level topology of generator and discriminator is a residual U-shaped network and a patch-based encoder, respectively.

The architecture of MGGAN is shown in Fig. 1, MGGAN consists of a multi-generator and a discriminator. The HE image of BC is the input of MGGAN. The multi-generator consists of two U-shaped networks with different scale [34], [35], there is a concatenation layer before outputting the transformed image. The encoder is the compression path that is composed of convolution and down-sampling operations. The decoder is the expansion path that consists of convolution and up-sampling. The input of each expanded network block is spliced by the features sampled on the previous layer and the features of the compression path. The discriminator's primary architecture is based on a patch-based encoder. A patch-based discriminator emphasizes high-frequency structures and limits attention to local patches of varying scales within an image. The concept behind the patch-based discriminator is to concentrate on high-frequency information within the original image. It accomplishes this by using patches of size N×N rather than analyzing the entire image to determine authenticity.

#### 2.2.1 Multi-generator architecture

The goal of I2I translates one image into another. Both two images have the same size and different surface appearance. This property is very consistent with the end-to-end U-shaped network. U-Net is a complete convolution structure proposed by the pattern recognition and image processing group of Freiburg University in Germany [36]. Compared with the common network with the encoder-decoder structure that is first down-sampling image to the low dimension and then up-sampling feature map to the original resolution, U-Net adds the skip connection operation and concatenates the encoder and decoder feature maps. The original U-Net is divided into two parts. The left side is the encoder part composed of convolution and down-sampling operations, the right side is the decoder part of convolution and up-sampling. The input of each decoder block is spliced by the features sampled on the previous layer and the features of the compressed path. Many previous U-Net-based methods have been used in I2I tasks [37], [38], [39]. These methods usually are very effective in improving details.

The encoder and decoder share a significant amount of low-level information, aiding in the reconstruction of object edge positions in the translated image. However, the diversity and sparsity of cell morphology and location in HE pose a challenge for the generator to accurately reconstruct cell locations. To overcome this obstacle, a multi-scale U-Net structure is utilized to construct the generator, as depicted in Fig. 1. The generator loss function is defined in Eq. (4).

(4) $$L_{cGAN}(G,D)=E_{x,y}[\log D(x,y)]E[\log(1-D(x,G(x)))].$$

#### 2.2.2 Patch GAN

**Figure 2:**
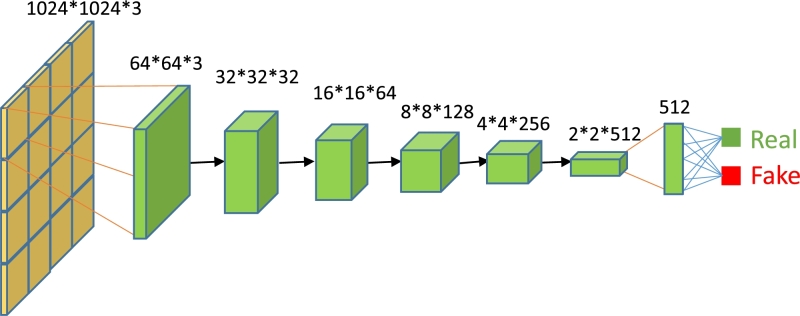
Overview of the patch-based discriminator architecture.

Using low-level information provided by the generator to reconstruct images is a challenging task. To overcome this issue, we have employed two strategies. The first involves the confrontation between the discriminator and generator to reconstruct high-level information, while the second uses L1-loss as part of the loss function. To improve the reconstruction of high-level information in MGGAN, we have used a patch-based discriminator. Our approach divides the whole image into independent patches of size N x N, and input them into the discriminator, which judges whether each patch of the image is true or false. The final discriminator output is the average of the results of all patches of the image. The implementation process is illustrated in Fig. 2. The translation map is divided into 16 independent patches, and features are extracted and reduced in dimensionality using a discriminator neural network. The probability that the translation map output is true is then calculated using the sigmoid activation function, the final loss is calculated using Binary Cross-Entropy Loss (BCEloss).

In addition, we employ L1-regularization-based loss to increase the similarity between the generated image and the training image. This approach not only enhances the clarity of the generated image but also assists GAN in generating the details of the translated image. The L1-based loss is defined in Eq. (5).

(5) $$L_{L1}(G)=E_{x,y}[{\left\|y-G(x)\right\|}_{1}].$$

The ultimate objective is to optimize the generator and discriminator to achieve the maximum and minimum game respectively. The overall objective function can be expressed in Eq. (6).

(6) $$G^{⁎}=argmin_{G}max_{D}L_{cGAN(G,D)}+\lambda L_{L1}(G)$$

Finally, we use cross-entropy loss to correct the consistency between the synthetic image (G(x)) and the real image (*x*). The cross-entropy loss $L_{cross}$ can be expressed in Eq. (7).

(7) $$L_{cross}=-\frac{1}{\frac{M}{2}-1}\sum_{i=0}^{\frac{M}{2}-1}x_{i}.log(G(x_{i}))+(1-x_{i}.log(1-G(x_{i}))),$$

where *M* denotes the image size.

The final objective loss function ($L_{final}$) (Eq. (8)) is defined as follows:

(8) $$L_{final}=argmin_{G}max_{D}L_{cGAN(G,D)}+\lambda_{1}L_{L1}(G)+\lambda_{2}L_{cross},$$

where $\lambda_{1}$ and $\lambda_{2}$ are the hyper-parameters employed to balance the final objective loss function.

## 3 Material and metrics

### 3.1 Material

**Figure 3:**
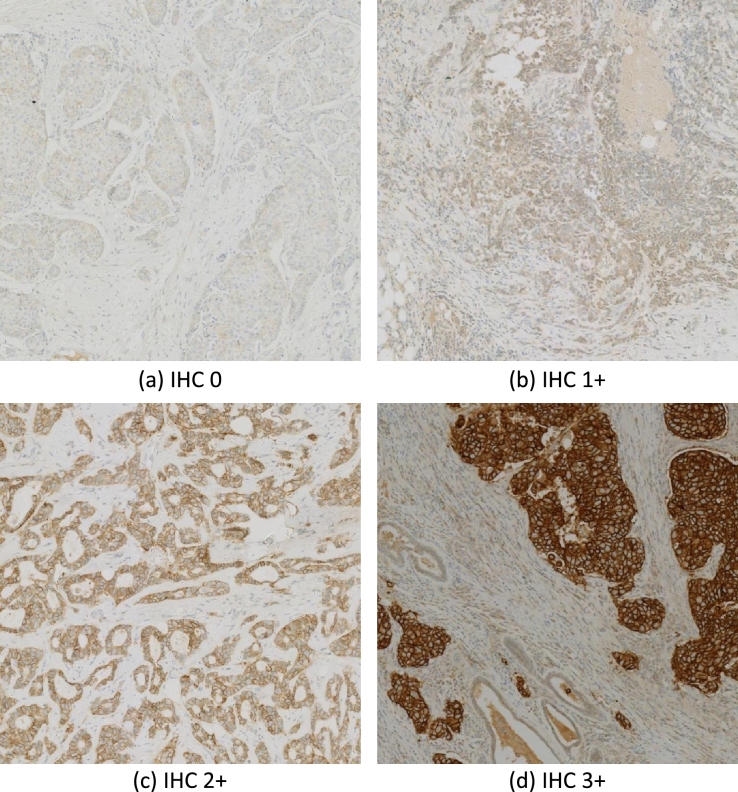
Visualization of 4 typical of IHC samples. IHC 0, unstained or weakly stained membrane and in ≤ 10% of tumor cells (Fig. 3 (a)); IHC 1+, incomplete membrane staining and in ≥ 10% of tumor cells (Fig. 3 (b)); IHC 2+, weak moderate complete membrane staining and in ≥ 10% of tumor cells (Fig. 3 (c)); IHC 3+, complete and intense staining and in ≥ 10% of tumor cells.

We conducted an evaluation of the proposed MMGAN on a public breast cancer challenge dataset, specifically the breast cancer immunohistochemical (BCI) image generation challenge [31]. The BCI dataset, which provides data for synthesizing IHC data from HE-stained images, can be downloaded from the BCI challenge website.^1^ The data was collected using a Hamamatsu NanoZommer S60 scanner with the following acquisition parameters: a scanning speed is 60 seconds and a resolution of 0.46 *μ* per pixel. The BCI dataset includes registered images, and any WSI pairs that can not be aligned were removed. It contains 4873 paired samples (9746 images), among of them 3896 pairs for training and 977 for testing. These samples are from HE staining WSI and corresponding immunohistochemical WSI pathological sections of 51 breast cancer patients. The IHC samples are divided into 4 kinds: 0, 1+, 2+, and 3+. Fig. 3 shows the 4 typical IHC images. The panel of 4 typical IHC images are labelled and the figure captions describe each panel are labelled and the figure captions describe each panel in Fig. 3.

### 3.2 Evaluation metrics

The BCI Challenge offers Peak Signal Noise Ratio (PSNR) and Structural Similarity (SSIM) as evaluation metrics for the assessing the quality of translated images. PSNR is an objective metric that measures image quality by calculating the error between the corresponding pixels of two images. PSNR is defined as follows:

(9) $$MSE=\frac{1}{m⁎n}\sum_{i=0}^{m-1}\sum_{j=0}^{n-1}{\left[I(i,j)-K(i,j)\right]}^{2},$$

where *MSE* stands for mean square error, *I* stands for noise free original image, *K* stands for noise image after adding noise to *I*, and m⁎n is the scale of IandK. Eq. (9) can be used to derive the formula for PSNR.

(10) $$PSNR=10\log_{10}(\frac{MAX_{I}^{2}}{MSE})=20\log_{10}(\frac{MAX_{I}}{\sqrt{MSE}}),$$

where $MAX_{I}$ is the maximum pixel value of the *I*. The higher the PSNR value, the better the image quality.

While PSNR (Eq. (10)) is an objective metric commonly used to evaluate image quality, it may not always reflect the level of quality as perceived by human eyes. To address this issue, the challenge also employs SSIM as an evaluation metric. SSIM is a full-reference image quality assessment metric that measures the similarity between two images in terms of brightness, contrast, and structure. The higher the value of SSIM, the better the quality of the image, with a maximum value of 1. The definition of SSIM is defined in Eq. (11).

(11) $$SSIM(I,K)=\frac{\left(2\mu_{i}\mu_{k}+c_{1})(2\sigma_{ik}+c_{2}\right)}{\left(\mu_{i}^{2}+\mu_{k}^{2}+c_{1})(\sigma_{i}^{2}+\sigma_{k}^{2}+c_{2}\right)}),$$

where $\mu_{i}$ and $\mu_{k}$ represent the mean values of image *I* and *K*, respectively. $\sigma_{i}$ and $\sigma_{k}$ represents the variance of image *I* and *K*, respectively, $\sigma_{ik}$ represents the covariance of image *I* and *K*. $c_{1}$ and $c_{2}$ are constants, in order to avoid the situation where the denominator is 0.

## 4 Experiments and results

### 4.1 Experimental setup

For training MGGAN, we utilized the Adam optimizer [40] with the parameters $\beta_{1}=0.5$ and $\beta_{2}=0.999$. The model was trained for 200 epochs, and the learning rate for both the generator and discriminator was set to 0.0001. We conduct experiments in Python software and run on a server with two NVIDIA 3090 GPUs. The training set was used for training and fine-turning the MGGAN model, the test set was used to verify the performance of MGGAN. For a fair comparison, all methods were trained and tested on the same set of data samples.

### 4.2 Influence of the hyper-parameters

**Figure 4:**
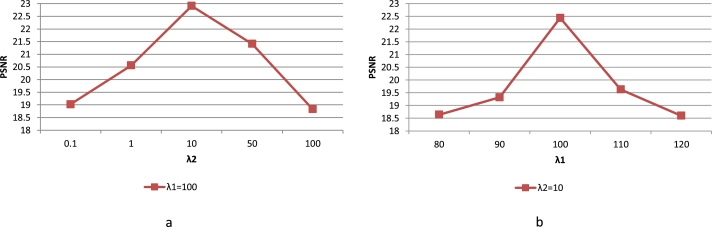
The influence of different hyper-parameters on PSNR. (a) displays the synthesis performance of MGGAN when the values of *λ*_2_ = 10. (b) displays the statistical result on PSNR metric when *λ*_1_ = 100.

To investigate the influence of the hyperparameters on the performance of the final MGGAN model and determine the optimal values, i.e., $\lambda_{1}$ and $\lambda_{2}$ in the final objective loss function Eq. (6). We performed a series of experiments on the training set (3896 samples) of the BCI dataset using ten-fold cross-validation. In selecting hyperparameters, we referred to parameter settings used in image synthesis methods based on GANs by other researchers [41], [42], [43]. Specifically, first, we fixed the interval of the hyper-parameters value, $\lambda_{1}\in [80,120]$ and $\lambda_{2}\in [0.1,100]$. Second, we fixed $\lambda_{1}=100$ and set value of $\lambda_{2}$ from 0.1 to 100. Finally, the statistical results in terms of PSNR are given in Fig. 4. Fig. 4 (a) displays the synthesis performance of MGGAN when the values of $\lambda_{2}=10$. As $\lambda_{1}$ varies from 80 to 100, the polyline of PSNR shows an upward trend. However, when $\lambda_{1}$ is greater than 100, PSNR shows a negative correlation trend. Fig. 4 (b) displays the statistical result on PSNR metric when $\lambda_{1}=100$. As observed, the polyline of PSNR shows an upward trend when $\lambda_{2}$ increases from 0.1 to 10. However, when $\lambda_{2}$ is greater than 10, PSNR shows a negative correlation trend. Based on the above analysis, we fixed $\lambda_{1}=100$ and $\lambda_{2}=10$, which is conducted in the following experiments.

### 4.3 Comparison with state-of-the-art methods

To evaluate the effectiveness of the proposed MGGAN method, we compare its performance with several other state-of-the-art translation methods on the BCI dataset. In all comparison methods, cycle GAN is trained using the unsupervised strategy, the other methods adopt the supervised training strategy. As shown in Table 1, MGGAN outperforms the other methods and achieves better results. Specifically, when compared to the unsupervised method (cycle GAN), our proposed MGGAN demonstrated a substantial improvement in PSNR, with an increase of 15.77 dB. Among the supervised translation methods, Pix2pix is a representative method of image translation, which plays a benchmark in our experiment. Three metrics of Pix2pix are significantly more advantageous than cycle GAN. However, among the supervised methods, Pix2pix has the worst performance. On the basis of inheriting the essence of Pix2pix, the performance of methods DRPANs, Pix2pixHD, SRGAN, CoCosNetv2, DP-GAN, and MGGAN is gradually improved. CoCosNetv2 is an improved method of CoCosNet [16], CoCosNet can use the fine texture in the sample to reduce the illusion of local texture, but it is difficult to use the global information to guide the network to translate the fine structure. CoCosNetv2 adopts the PatchMatch iteration method to realize the full-resolution semantic correspondence, which promotes image translation based on samples. Our proposed MGGAN outperforms the other supervised translation methods in all three metrics. Specifically, compared with DP-GAN which obtains the second-best performance, MGGAN boosts the quality of the translation image with PSNR rising from 30.01 to 31.78, SSIM rising from 0.47 to 0.48, and MSE dropping from 1.67e-3 to 1.59e-3. Compared with CoCosNetv2 and DP-GAN, MGGAN also adopts the idea of obtaining global semantic information from patches and prime direction loss, and antagonism loss. In addition, we also introduce the spectral regularization term ($L_{cross}$) to eliminate high-frequency distortion.

**Table 1:** Comparison results of the eight methods on the test set. The best results are shown in bold.

Methods	PSNR(dB)	SSIM	MSE
Cycle GAN [22]	16.01	0.36	3.06e-3
Pix2pix [15]	18.72	0.40	2.62e-3
DRPANs [23]	19.28	0.42	2.25e-3
Pix2pixHD [24]	20.07	0.45	1.86e-3
SRGAN [25]	23.96	0.45	1.89e-3
CoCosNetv2 [28]	29.17	0.47	1.60e-3
DP-GAN [44]	30.01	0.47	1.62e-3
MGGAN^⋆^	25.07	0.46	1.73e-3
MGGAN	**31.78**	**0.48**	**1.59e-3**

In addition, we proposed MGGAN^⋆^ model that retains the structure of the MGGAN and abandoned the cross-entropy loss function. As shown in Table 1, the performance of method MGGAN^⋆^ is significantly inferior to that of method MGGAN. This indicates that the cross entropy loss used in our loss function as the regularization term can ensure consistency between the synthesized image and the real image.

### 4.4 Visualization of render

**Figure 5:**
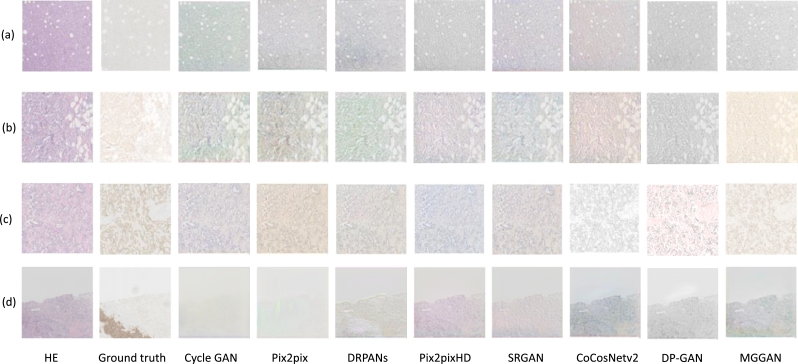
Visualization of different methods on four type IHC images. Fig. 5(a) displays the translation results of an IHC0 image. Fig. 5(b) displays the translation results of an IHC1+ image. Fig. 5(c) displays the translation results of an IHC2+ image. Fig. 5(d) displays the translation results of an IHC+3 image.

We selected four representative BCI samples to visually demonstrate the comparison results, as shown in Fig. 5. Specifically, we selected four samples to illustrate the synthetic results for four types of IHC images. Our proposed MGGAN method outperforms the other comparison methods in terms of authenticity, as can be observed in the visual results. Overall, the results of our MGGAN method are superior to the other comparison methods in terms of authenticity and visual quality. In IHC0 and IHC1+ image translation results, the difference between the image generated by all methods and the ground reality is very small (Fig. 5 (a) (b)); For IHC2+ samples, the image generated by CoCosNetv2, DP-GAN, and MGGAN is better than other methods, and the image generated by MGGAN is better than that of DP-GAN and CoCosNetv2. The image generated by our method is more similar to the ground reality, and the image ambiguities of other comparison methods translation are less blurry (Fig. 5 (c)); However, all methods struggled to accurately translate the IHC3+ samples and identify the dark regions with high expression of HER2 (Fig. 5 (d)). This issue may be attributed to the imbalance of IHC3+ samples and image pixel distribution, which requires further investigation in future studies. In summary, both the qualitative and quantitative experimental results of Table 1 and Fig. 5 demonstrate that our MGGAN method achieves the closest translation to the ground truth among state-of-the-art methods. In addition, there is still a need to explore more effective ways to enhance the accuracy of IHC3+ image translation.

### 4.5 Ablation studies

**Figure 6:**
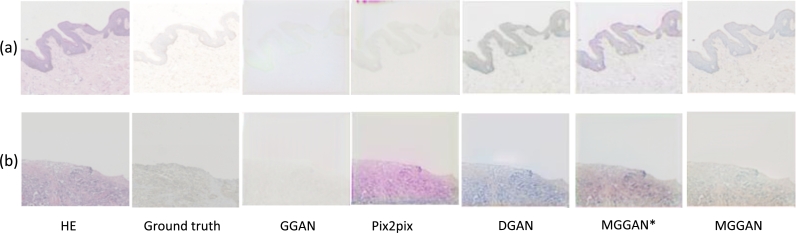
Visualization of ablation studies. (a) denotes the results of an IHC+2 image and (b) denotes the results of IHC+3 image.

We conduct ablation experiments to evaluate the contribution of each key component in our proposed MGGAN, including the multi-generator, the patch-based discriminator, and the correct regulation term ($L_{cross}$). Pix2pix method is the baseline of our ablation experiment, the details of experiments and results are shown in Table 2. In addition, we visualize an IHC2+ image and an IHC+3 image in Fig. 6 (a) and (b).

**Table 2:** Comparison results of the ablation experiment on the test set. The best results are shown in bold.

Methods	PSNR(dB)	SSIM	MSE
GGAN	18.58	0.39	2.46e-3
Pix2pix	18.72	0.40	2.62e-3
DGAN	26.91	0.44	1.95e-3
MGGAN	**31.78**	**0.48**	**1.59e-3**

To evaluate the effectiveness of the multi-generator in our proposed method, we compare the baseline method, Pix2pix, with the signal generator GAN (GGAN). In the GGAN method, we only use the signal generator, keeping the patch-based discriminator and without $L_{cross}$. In MGGAN^⋆^, we retained the structure of the proposed model and abandoned the cross-entropy loss function. As shown in Table 2, compared to the baseline method, the GGAN method reduces the performance by 0.14 dB PSNR, 0.01 SSIM, and 0.16e-3 MSE on the test samples. In addition, as shown in Fig. 6, the translated image by GGAN is fuzzier and has low recognition. Both comparison results indicate that the multi-generator and $L_{cross}$ are effective in image translation.

To evaluate the contribution of the patch-based discriminator in our proposed method, we compare Pix2pix with the GAN (DGAN) with the multi-generator and patch-based discriminator, without $L_{cross}$. As shown in Table 2, the quantitative results of DGAN improve the performance by 8.19dB PSNR, 0.04 SSIM, and 0.57e-3 MSE on the test samples. The patch-based discriminator contributes significantly to the improved performance of the DGAN method. Consistent with the quantitative results, Fig. 6 shows that the images generated by DGAN are more similar to the ground truth than those generated by Pix2pix.

In addition, to investigate the effectiveness of the $L_{cross}$ in our proposed method, we compare the final method MGGAN with DGAN. As shown in the last two rows in Table 2, with the $L_{cross}$, the average metrics of MGGAN further increase by 4.87dB PSNR, 0.04 SSIM, and 0.3e-3 MSE on the test samples. According to these metrics, we can infer that the $L_{cross}$ is effective in improving our method performance. Furthermore, although MGGAN^⋆^ and MGGAN have the same structure, the performance of MGGAN^⋆^ is significantly inferior to that of MGGAN. This indicates that the cross-entropy loss used in our loss function as the regularization term can ensure consistency between the synthesized image and the real image. Notably, compared with the baseline method Pix2pix, our proposed method shows obvious advantages, indicating that the effective combination of three key components can further improve the quality of image generation. This advantage is also shown in Fig. 6. The generated graph of our proposed method is closer to the ground truth.

Overall, the quantitative and qualitative experimental results suggest that each key component of our method plays a significant role in improving the performance of the baseline network. Effectively combining these components can further enhance the quality of image translation.

## 5 Discussion

In our study, we propose MGGAN, an end-to-end structure for translating HE images into IHC images. This translation can provide an important means for evaluating diagnostic markers using the cheap and easily available HE images. Considering the differences between HE and IHC images, MGGAN leverages the low-frequency and high-frequency components of the image to complement each other, resulting in improved HE image details translation. Furthermore, MGGAN utilizes a double U-shaped architecture generator to address the low-frequency components and a patch-based discriminator to tackle high-frequency components. Additionally, we include the cross-entropy loss as the correct regularization term in the loss function to ensure consistency between the synthetic and real images. Experimental results demonstrate that these strategies effectively improve the quality of the synthesized image.

To evaluate the advantages of our proposed method, we used statistical and visual methods for both quantitative and qualitative comparison. We utilized PSNR, SSIM, and MSE as evaluation indicators to assess the MGGAN method on the BCI dataset. To demonstrate the superiority of MGGAN, we compared it with seven other advanced GAN image synthesis methods. As shown in Table 1, our proposed MGGAN outperformed the other methods. Additionally, from the visual analysis in Fig. 5, we observed that the MGGAN method improved the quality of synthesis visually. The multi-generator provides more and richer contextual semantic information for our model, which is conducive to the synthesis of organizational details. Furthermore, the correct regulation term in the loss function ensures consistency between the synthetic and real images. We have verified the positive role of this strategy in the analysis of ablation experiments in Section 4.5. We also evaluated the effectiveness of the three key components in the ablation experiment, including the multi-generator, the patch-based discriminator, and the correct regulation term. Both the quantitative and qualitative experimental results suggest that each key component of our method is paramount to the performance improvement of MGGAN, and effectively combining them can further enhance the image translation quality.

Although our current model has achieved considerable performance, there are still some limitations. Firstly, the limited sample size limits the performance of neural networks. Although data augmentation can alleviate this limitation, adding a large number of new samples can further improve the performance of neural networks. In our future work, we will focus on researching larger sample datasets to further improve our image synthesis capabilities. Secondly, the singularity of input data types makes it difficult for the model to obtain additional information. To address this issue, we will explore combining multi-modal data to improve synthesis accuracy. Thirdly, these methods may be difficult to identify dark regions with high HER2 expression. Therefore, we will also study the effectiveness of our comprehensive image synthesis results in future work.

## 6 Conclusions

In this study, we propose an end-to-end GAN-based image translation architecture aimed at narrowing the gap between synthetic and real images. Our approach employs a multi-generator to enhance the semantic information and introduce a cross-entropy loss as a regularization term to improve the consistency between the synthetic image and the real image. Our experimental and visualization results indicate that our method outperforms other most advanced methods in terms of both quantitative and qualitative aspects. Compared with traditional IHC staining techniques, our research has the advantages of low cost and high efficiency, providing new ideas for IHC staining.

## CRediT authorship contribution statement

**Liangliang Liu:** Writing – review & editing, Writing – original draft, Methodology, Investigation. **Zhihong Liu:** Methodology, Formal analysis. **Jing Chang:** Formal analysis, Data curation. **Hongbo Qiao:** Resources, Formal analysis. **Tong Sun:** Software, Conceptualization. **Junping Shang:** Writing – review & editing, Investigation.

## Declaration of Competing Interest

The authors declare that they have no known competing financial interests or personal relationships that could have appeared to influence the work reported in this paper.

## Data Availability

The dataset is a public dataset that comes from the breast cancer immunohistochemical (BCI) image generation challenge.

## Footnotes

1 https://bci.grand-challenge.org/.
